# Lymphoepithelial Carcinoma of the Breast Treated With Partial Mastectomy and Sentinel Lymph Node Biopsy

**DOI:** 10.7759/cureus.39190

**Published:** 2023-05-18

**Authors:** Kristen Lentsch, Leah C Dauterman, Betty Fan

**Affiliations:** 1 Surgery, Indiana University School of Medicine, Indianapolis, USA; 2 Medical Education, Indiana University School of Medicine, Indianapolis, USA; 3 Surgical Oncology, Indiana University School of Medicine, Indianapolis, USA

**Keywords:** sentinel lymph node biopsy, breast, lymphoepithelial carcinoma, partial mastectomy, breast cancer

## Abstract

Lymphoepithelial carcinoma of the breast (LELC) is a rarely encountered form of breast carcinoma, and there is limited information treatment for this entity. We present a case of a 55-year-old postmenopausal female presenting with a left breast mass on screening mammogram with core needle biopsy showing lymphoepithelial carcinoma. The patient was treated with surgical resection of the mass and sentinel lymph node biopsy, followed by adjuvant chemotherapy and radiation. Given the rarity of this type of breast carcinoma, our case study continues to add to the treatment considerations in the literature, specifically the role of sentinel lymph node.

## Introduction

Lymphoepithelial carcinoma of the breast, also known as lymphoepithelioma-like carcinoma of the breast (LELC), is a rarely encountered form of breast carcinoma. It has been described exclusively in case reports and limited series. LELC may also be found in the skin, salivary glands, thyroid gland, thymus, lung, female genital system, gastrointestinal system, and urinary system [1]. Although the nasopharyngeal tumors are more aggressive, tumors from other sites have also demonstrated an ability to metastasize through local, hematogenous, and lymphatic means [1].

Histologically, LELC of the breast is described similarly to lymphoepithelioma of the nasopharynx [2]. It is characterized by its multinodular structure, prominent nucleoli, and dense mononuclear infiltrate [3]. Unlike lymphoepithelioma carcinoma of the nasopharynx, numerous case reports have demonstrated that LELC of the breast is not associated with Epstein-Barr virus (EBV) infection [3,4]. Some limited research suggests that LELC of the breast may be associated with human papillomavirus (HPV) infection, but there is limited research to support this [5,6].

LELC of the breast typically presents as a triple-negative tumor, but it has been demonstrated to have a better prognosis than other triple-negative breast cancers. For this reason, it has frequently been compared to medullary carcinoma of the breast [7].

Due to the rarity of LELC of the breast, no standardized treatment consensus is available. Of the documented cases of LELC of the breast, all have been managed surgically with local excision, quadrantectomy, or mastectomy [1]. Lymph node staging for LELC in the breast has not been standardized given the scarcity of this entity. Most patients also receive adjuvant radiotherapy and/or chemotherapy in addition to surgical resection [7]. We present our case to add to the literature regarding management considerations of this unusual breast carcinoma.

## Case presentation

A 55-year-old postmenopausal female was found to have a suspicious mass in her left breast detected by screening mammography. The patient reported a six-month history of mild breast sensitivity but denied any other changes in her breast. The patient had received cervical Pap screening within the same year, which was negative for all forms of HPV. The patient’s family history was notable for breast cancer in her mother at age 63 and ovarian cancer in her maternal grandmother.

**Figure 1:**
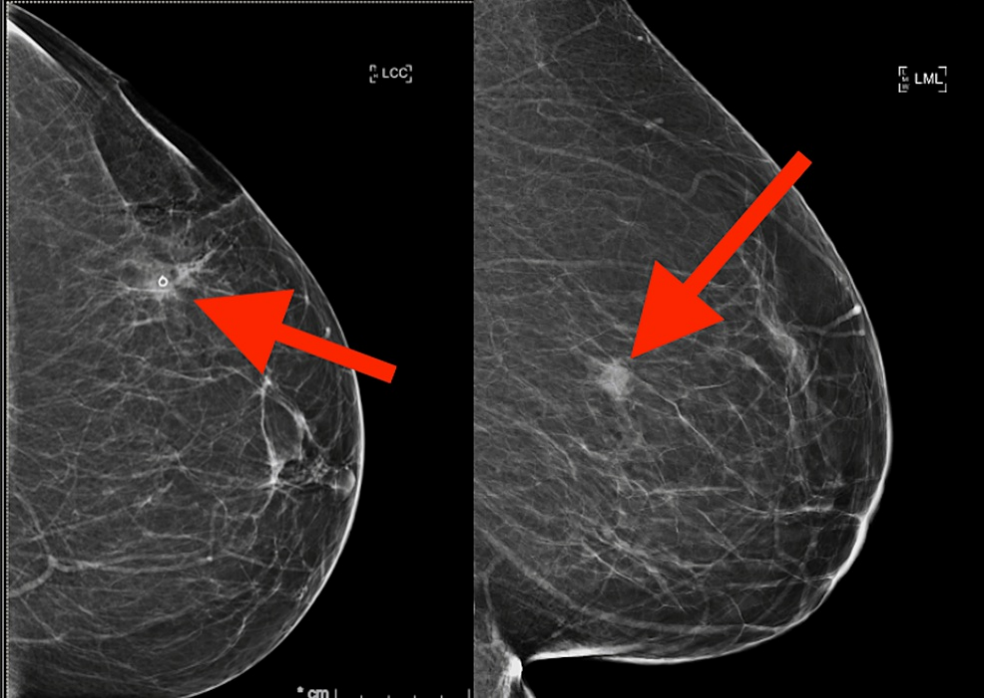
Mammogram of the left breast demonstrating a mass containing a biopsy clip in the upper outer quadrant after core needle biopsy.

**Figure 2:**
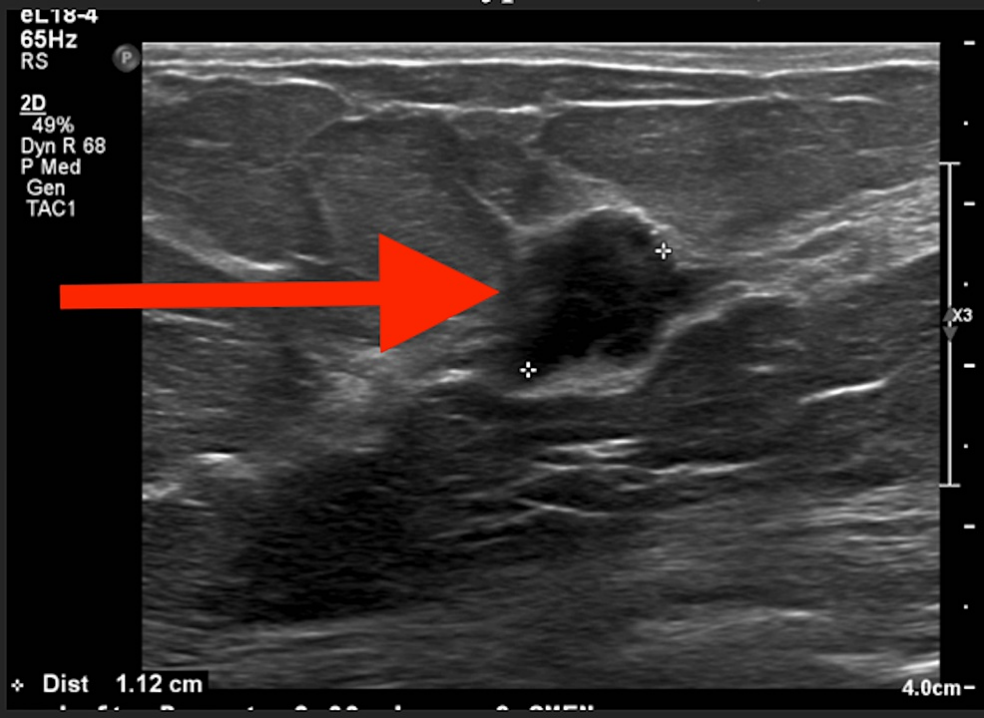
Ultrasound of the left breast showing a hypoechoic mass, measuring 1.1 cm in diameter, with irregular borders.

A diagnostic mammogram following the screening mammogram revealed a persistent mass with indistinct margins in the left breast (Figure 1). Ultrasound revealed a 1.1-cm hypoechoic mass with irregular borders in the upper outer quadrant of the left breast at the 3 o'clock position, 9 cm from the nipple, which correlated with the mammographic mass of concern. All visualized axillary lymph nodes were unremarkable (Figure 2).

**Figure 3:**
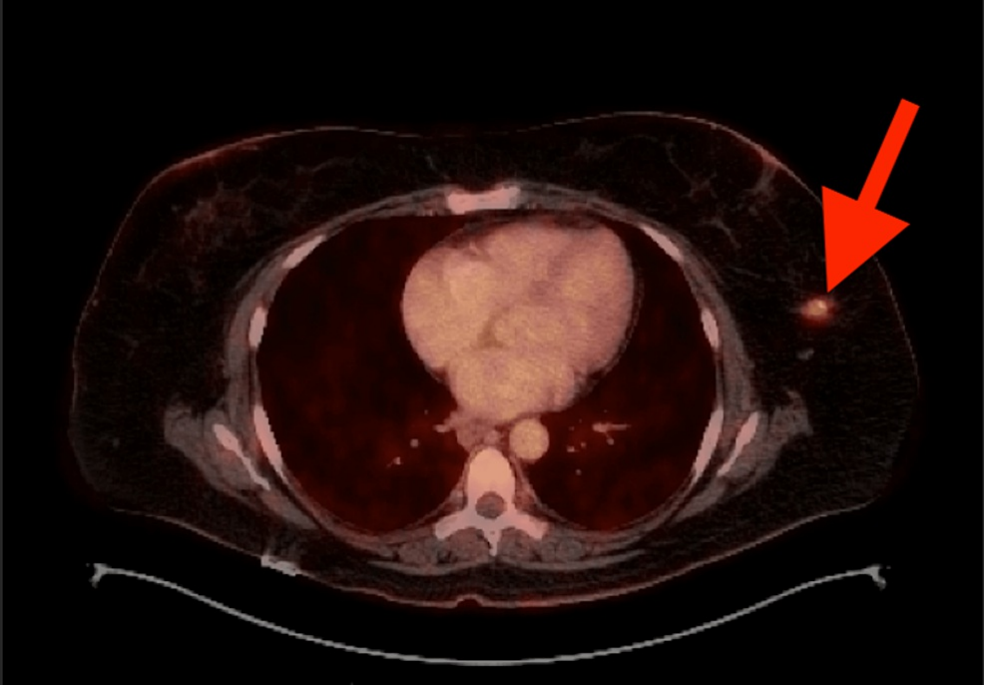
PET scan demonstrating a solitary FDG avid lesion in the left breast, measuring 1.6 cm in diameter. There was no evidence of regional adenopathy or distant metastatic disease. PET, positron emission tomography; FDG, fluorodeoxyglucose

Core needle biopsy of the breast mass revealed an ER- PR- Her2- poorly differentiated epithelial proliferation with large, vesicular nuclei, prominent nucleoli, and intermixed lymphoblastic cells. It was determined that the mass was consistent with EBV-negative lymphoepithelial carcinoma. Given that lymphoepithelial carcinoma rarely originates from the breast, it was unclear whether the mass represented a primary or secondary lesion. Therefore, a positron emission tomography/computed tomography (PET/CT) was performed, which demonstrated no distant metastases or other primary malignancies (Figure 3).

The patient’s case was reviewed at our institution’s multidisciplinary breast conference, and decision was made to proceed with surgical resection and staging, similar to breast carcinoma management, and adjuvant therapies as indicated. Neoadjuvant chemotherapy was not pursued given the small primary tumor and clinically negative lymph node examination.

The patient opted for breast conservation surgery with a partial mastectomy and sentinel lymph node biopsy. Final surgical pathology following resection demonstrated a triple-negative, EBV-negative, high-grade carcinoma, which was ultimately determined to be LELC. Sentinel lymph node biopsy was negative for malignancy.

Her treatment was then followed by adjuvant chemotherapy with doxorubicin and cyclophosphamide for four cycles and paclitaxel weekly for 12 cycles. Whole breast radiation with a boost to the tumor bed also followed. The patient overall tolerated her adjuvant therapies well except for some mild transaminitis, fatigue, and diarrhea, requiring a reduction during her paclitaxel cycles. She recently presented for her six-month follow-up and is doing well with no evidence of disease recurrence or metastasis.

## Discussion

This case report presents a rare form of breast cancer for which no current clinical management guidelines exist. There have been fewer than 40 cases described in the literature, of which all have received surgical resection with various combinations of lymph node dissection or biopsy, chemotherapy, and/or radiation therapy.

In our case, sentinel lymph node biopsy was offered to the patient in combination with partial mastectomy, despite a lack of standardization on the use of sentinel lymph node biopsy in the surgical management of LELC of the breast. In this patient specifically, the tumor was small (<2 cm), PET/CT demonstrated no signs of metastasis, and no pathologically enlarged lymph nodes were encountered during the operation. For these reasons, sentinel lymph node biopsy was chosen over axillary lymph node dissection (ALND) based on multidisciplinary discussion and consensus to proceed with staging similar to comparable breast carcinomas.

Of note, one review of the literature analyzed the treatment of 16 cases of LELC of the breast where all 16 patients received either mastectomy, quadrantectomy, or local excision, based on lesion size and patient or surgeon preference. Of these cases, 14/16 underwent ALND, with 28.6% (4/14) of those cases demonstrating at least one lymph node, which was positive for malignancy [1]. In another more recent literature review examining 22 cases of LELC of the breast, nodal involvement was reported in 22% (5/22) of cases, and no distant metastases were reported [8]. Contrary, sentinel lymph node biopsy has also been used in the clinical management of LELC of the breast, with three case studies reporting disease-free outcomes following its use [6,9,10]. As such, the usage of sentinel lymph node biopsy in LELC should be strongly considered in modern-day management instead of ALND given the relatively low incidence of axillary involvement in LELC and the known reliability of sentinel lymph node technique in the management of breast cancers.

The use of chemotherapy and radiotherapy for LELC of the breast has also been quite variable. The most recent literature review reported that 58% of reviewed cases received some form of adjuvant chemotherapy, 12% received radiation therapy alone, and 15% received a combination of chemotherapy and radiation therapy [7]. Few reported cases utilized neoadjuvant chemotherapy, with only 1/33 patient in Nieto-Coronel et al.’s literature review having completed a course of taxotere, adriamycin, and cyclophosphamide prior to surgical resection.

Although LELC of the breast is most commonly a triple-negative tumor, the prognosis is often quite favorable [11]. Of the 33 cases reviewed in Nieto-Coronel et al.’s review, 9% (3/33) experienced disease recurrence, 3% (1/33) experienced metastasis of disease, and no patients died of the disease within an average of 31 months following surgical resection [7].

Given the rare nature of this form of breast cancer, best practices for surgical management of LELC are yet to be determined. However, based on current literature and as seen in our case study, surgical resection of the mass with sentinel lymph node biopsy staging and adjuvant therapy (as indicated) is a reasonable approach in early stage LELC, comparable to standard management practices in more common types of breast cancer.

## Conclusions

LELC is a rare clinical entity, with scant research available to assist with clinical decision-making. The use of chemotherapy and radiation therapy to treat LELC of the breast is quite variable, with the majority of patients receiving some form of adjuvant chemotherapy. Given the rarity of LELC in the breast, best practice currently suggests treating this entity with multidisciplinary input and extrapolation from breast carcinoma treatment guidelines. Staging for LELC of the breast may be successfully performed by a sentinel lymph node biopsy until more data are available. ALND may be an overtreatment in clinically node-negative LELC cases of the breast, and sentinel lymph node biopsy is an acceptable alternative.

## References

[1] Dinniwell R, Hanna WM, Mashhour M, Saad RS, Czarnota GJ (2012). Lymphoepithelioma-like carcinoma of the breast: a diagnostic and therapeutic challenge. Curr Oncol.

[2] Thompson LD, Whaley RD (2021). Lymphoepithelial carcinoma of salivary glands. Surg Pathol Clin.

[3] Dadmanesh F, Peterse JL, Sapino A, Fonelli A, Eusebi V (2001). Lymphoepithelioma-like carcinoma of the breast: lack of evidence of Epstein-Barr virus infection. Histopathology.

[4] Kijima Y, Hokita S, Takao S (2001). Epstein-Barr virus involvement is mainly restricted to lymphoepithelial type of gastric carcinoma among various epithelial neoplasms. J Med Virol.

[5] Kulka J, Kovalszky I, Svastics E, Berta M, Füle T (2008). Lymphoepithelioma-like carcinoma of the breast: not Epstein-Barr virus-, but human papilloma virus-positive. Hum Pathol.

[6] Nio Y, Tsuboi K, Tamaoki M, Tamaoki M, Maruyama R (2012). Lymphoepithelioma-like carcinoma of the breast: a case report with a special analysis of an association with human papilloma virus. Anticancer Res.

[7] Nieto-Coronel MT, Perez-Sanchez VM, Salazar-Campos JE, Diaz-Molina R, Arce-Salinas CH (2019). Lymphoepithelioma-like carcinoma of breast: a case report and review of the literature. Indian J Pathol Microbiol.

[8] Fadila K, Faycal A, Lamiaa J, Mohammed B, Nabil I (2019). Lymphoepithelioma-like carcinoma of the breast: a case report and review of the literature. Pan Afr Med J.

[9] O'Sullivan-Mejia E, Idowu MO, Davis Masssey H, Cardenosa G, Grimes MM (2009). Lymphoepithelioma-like carcinoma of the breast: diagnosis by core needle biopsy. Breast J.

[10] Trihia H, Siatra H, Gklisty H, Diamantopoulos P, Arapantoni-Dadiotis P, Kalogerakos K (2012). Lymphoepithelioma-like carcinoma of the breast: cytological and histological features and review of the literature. Acta Cytol.

[11] Shet T, Pai T, Shetty O, Desai S (2016). Lymphoepithelioma-like carcinoma of breast-evaluation for Epstein-Barr virus-encoded RNA, human papillomavirus, and markers of basal cell differentiation. Ann Diagn Pathol.

